# The effect of BIS-guided sevoflurane anesthesia on postoperative nausea and vomiting in pediatric adenoidectomy: a prospective randomized controlled trial

**DOI:** 10.1590/1516-3180.2026.3631.13052026

**Published:** 2026-08-31

**Authors:** Engin Cetin, Ayse Sencan, Ahmet Yuksek, Bedirhan Gunel, Fatihhan Zeytun, Mehmet Yilmaz

**Affiliations:** I Department of Anesthesiology and Reanimation, University of Health Sciences Kocaeli City Hospital, İzmit, Kocaeli, Türkiye.; II Department of Anesthesiology and Reanimation, University of Health Sciences Kocaeli City Hospital, İzmit, Kocaeli, Türkiye.; III Department of Anesthesiology and Reanimation, University of Health Sciences Kocaeli City Hospital, İzmit, Kocaeli, Türkiye.; IV Department of Anesthesiology and Reanimation, University of Health Sciences Kocaeli City Hospital, İzmit, Kocaeli, Türkiye.; V Department of Anesthesiology and Reanimation, University of Health Sciences Kocaeli City Hospital, İzmit, Kocaeli, Türkiye.; VI Department of Anesthesiology and Reanimation, University of Health Sciences Kocaeli City Hospital, İzmit, Kocaeli, Türkiye.

**Keywords:** Monitoring, Intraoperative, Postoperative Nausea and Vomiting, Pediatric Anesthesia, Sevoflurane, Adenoidectomy, BIS-Guided Anesthesia, Pediatric Adenoidectomy, Postoperative Recovery, Emergence Time, Volatile Anesthetic Consumption

## Abstract

**BACKGROUND:**

Postoperative nausea and vomiting (PONV) is a frequent complication in pediatric patients undergoing adenoidectomy. Bispectral index (BIS) monitoring, an electroencephalography-based method for assessing anesthetic depth, enables individualized sevoflurane titration and may reduce unnecessary exposure to volatile anesthetics.

**OBJECTIVES:**

To determine whether BIS-guided sevoflurane anesthesia reduces PONV in children undergoing adenoidectomy.

**DESIGN AND SETTING:**

Single-center, prospective, randomized, assessor-blinded trial conducted at University of Health Sciences, Kocaeli City Hospital, Kocaeli, Türkiye.

**METHODS:**

Fifty-eight children aged 3–8 years (American Society of Anesthesiologists score I–II) undergoing elective adenoidectomy were randomized to BIS-guided anesthesia (n = 29) or standard care (n = 29). Both groups received sevoflurane anesthesia. The primary outcome was PONV within the first 24 postoperative hours. Secondary outcomes were emergence time, total sevoflurane consumption, remifentanil dose, and rescue antiemetic use.

**RESULTS:**

PONV within 24 hours occurred less frequently in the BIS-guided group than in controls (p = 0.015), with the greatest between-group differences observed at 0–1 hour and 1–6 hours. Sevoflurane consumption and emergence time were lower in the BIS-guided group (p < 0.05). BIS-guided anesthesia was independently associated with lower odds of PONV (OR = 0.212; 95% CI: 0.067–0.675; p = 0.009).

**CONCLUSIONS:**

BIS monitoring may be a useful strategy for optimizing anesthetic depth and reducing early PONV in pediatric patients.

**CLINICAL TRIAL REGISTRATION:**

ClinicalTrials.gov (NCT07189845). Available at: https://clinicaltrials.gov/study/NCT07189845.

## INTRODUCTION

Postoperative nausea and vomiting (PONV) is among the most common postoperative complications, with an overall incidence of approximately 30%.^1^ In pediatric patients, particularly following otorhinolaryngologic surgeries, this incidence may reach as high as 73%.^2^ Complications associated with PONV include dehydration, electrolyte imbalance, aspiration, and postoperative bleeding.^2^ In addition, PONV is associated with lower patient satisfaction and prolonged length of stay in the post-anesthesia care unit (PACU).^1,3^ Adenotonsillectomy and strabismus surgery have been reported to be the surgical procedures most frequently associated with PONV in children.^4,5^ Other factors associated with the development of PONV include surgical duration exceeding 30 minutes, a positive family history, the use of inhalational anesthetics, nitrous oxide, and opioids.^4-7^

The pharmacological effects of general anesthetics in children vary according to age and body surface area,^8^ which may lead to excessive or insufficient anesthetic doses.^8^ During inhalational anesthesia, anesthetic depth is commonly monitored using the minimum alveolar concentration (MAC).^9^ However, MAC-based monitoring may not accurately reflect the hypnotic component of anesthesia.^9^ Monitoring anesthetic depth using the bispectral index (BIS) allows more precise and individualized titration of anesthetic agents.^10^ BIS-guided anesthesia improves postoperative outcomes and reduces anesthetic drug consumption.^10,11^

## OBJECTIVES

The aim of this study was to evaluate the effect of BIS-guided sevoflurane anesthesia on PONV in pediatric patients undergoing adenoidectomy. We hypothesized that BIS-guided sevoflurane anesthesia would reduce the incidence of PONV within the first 24 postoperative hours.

## METHODS

### Study design and ethics

This study was a single-center, prospective, randomized, assessor-blinded clinical trial conducted at University of Health Sciences, Kocaeli City Hospital, in accordance with the principles of the Declaration of Helsinki and Good Clinical Practice. The design, conduct, and reporting of the study complied with the CONSORT 2025 (Consolidated Standards of Reporting Trials) guidelines.^12^

The study protocol was approved by the Kocaeli University Clinical Research Ethics Committee (Approval No. KAEK/12.bI.06; date of approval: July 17, 2025). Written informed consent was obtained from the parents or legal guardians of all participants. The trial was prospectively registered in the ClinicalTrials.gov database on September 2, 2025 (ClinicalTrials.gov Identifier: NCT07189845).

### Participants

Pediatric patients aged 3–8 years with American Society of Anesthesiologists (ASA) physical status I–II who were scheduled for elective adenoidectomy were included in the study. The exclusion criteria were a history of PONV, preoperative use of antiemetics, steroids, or sedative medications, acute upper respiratory tract infection, history of neurological or psychiatric disorders, body mass index below the 5th percentile or above the 95th percentile for age, and anatomical airway abnormalities.

### Patient flow, randomization, and blinding

A total of 62 patients were assessed for eligibility. Four patients were excluded before randomization because intravenous access could not be established during induction or because deviation from the standard anesthesia induction protocol was required. These protocol deviations resulted in exclusion before randomization.

**Figure 1:**
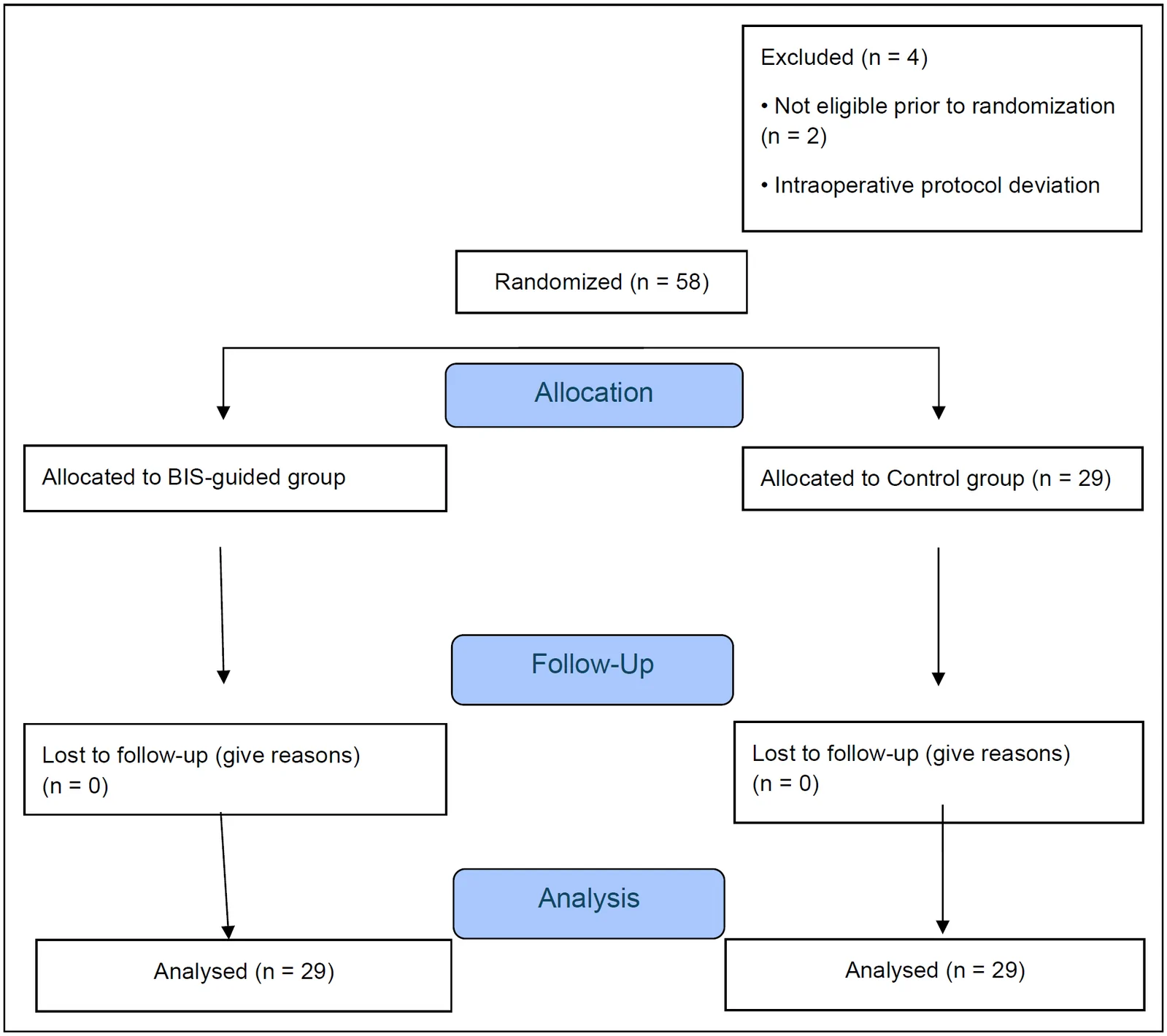
CONSORT flow diagram of patient enrollment and follow-up.

The remaining 58 patients were randomly assigned in a 1:1 ratio to two groups using a computer-generated block randomization method with a block size of four. Accordingly, 29 patients were allocated to the BIS-guided group and 29 patients to the control group. Group allocation was concealed using sequentially numbered, opaque, sealed envelopes prepared by an independent researcher not involved in the study process (Figure 1). Patients and investigators were blinded to group allocation. Blinding of the attending anesthesiologists was not feasible due to the nature of the BIS-guided anesthesia protocol, which may have introduced performance bias.

### Anesthesia protocol

Patients were transferred to the operating room after intravenous access had been established, and anesthesia induction was initiated intravenously. Intravenous fentanyl (1 μg/kg) was administered for analgesia, and anesthesia induction was achieved with propofol (2 mg/kg). Rocuronium (0.6 mg/kg) was administered to facilitate neuromuscular blockade and tracheal intubation.

Following endotracheal intubation, anesthesia was maintained with sevoflurane in an air/oxygen mixture. Mechanical ventilation was applied in pressure-controlled ventilation mode, with tidal volumes set at 6–8 mL/kg, respiratory rate adjusted to maintain end-tidal CO2 between 35 and 40 mmHg, and fresh gas flow set at 2 L/min. Intraoperative analgesia was provided with a remifentanil infusion titrated according to hemodynamic responses.

In the BIS-guided group, the sevoflurane concentration was titrated during maintenance of anesthesia to maintain a BIS value between 45 and 60. If BIS values exceeded the target range (> 60), the sevoflurane concentration was increased incrementally, whereas values below the target range (< 45) prompted a gradual reduction in sevoflurane concentration.

In the control group, the sevoflurane concentration was adjusted according to standard clinical practice to maintain age-adjusted MAC values between 1 and 1.3 and to keep hemodynamic parameters (heart rate and blood pressure) within ± 20% of baseline values. When MAC or hemodynamic targets were exceeded, anesthetic depth was adjusted by modifying the sevoflurane concentration.

### Measurement of anesthetic consumption

Total sevoflurane consumption (mL) during surgery was obtained from anesthesia machine records, and total remifentanil dose (μg/kg) was recorded from the infusion pump and anesthesia charts. These variables were analyzed as potential mediators of the effect of BIS-guided anesthesia on PONV.

Standard intraoperative monitoring included continuous assessment of heart rate, noninvasive blood pressure, peripheral oxygen saturation, end-tidal CO_2_, and body temperature throughout the procedure. BIS monitoring was performed using a BIS Vista™ monitor (Medtronic, Minneapolis). Anesthesia was delivered using an anesthesia workstation (Aisys™ Carestation, GE Healthcare, Madison).

### Emergence time

Emergence time was defined as the interval between discontinuation of sevoflurane at the end of surgery and eye opening in response to verbal commands. Neuromuscular blockade was reversed at the end of surgery with intravenous sugammadex (2 mg/kg). Emergence time was measured using a stopwatch and recorded in minutes. In cases where eye opening could not be assessed, limb movement or the onset of adequate spontaneous respiration was used as the emergence criterion.

### Outcome measures

#### Primary outcome

The primary outcome was the incidence of PONV within the first 24 postoperative hours.

#### Secondary outcomes

Secondary outcomes included emergence time, total sevoflurane consumption, total remifentanil dose, and the requirement for antiemetic therapy within the first 24 postoperative hours.

### Assessment of PONV

PONV was assessed at predefined time intervals after extubation. In the PACU, assessments were performed during the first 0–1 hour after extubation. On the ward, PONV assessments were conducted during the 1–6 hour, 6–12 hour, and 12–24 hour postoperative periods.

The severity of PONV was evaluated using a predefined four-point ordinal scoring system: score 0 indicated no nausea or vomiting; score 1 indicated mild nausea; score 2 indicated severe nausea or a single episode of vomiting; and score 3 indicated severe nausea accompanied by multiple episodes of vomiting or refractory PONV. Assessments were based on clinical observation and nursing reports.^13^ Rescue antiemetic treatment was administered when patients experienced vomiting or when the PONV score was ≥ 2.

The time points were defined as follows: the first postoperative hour in the PACU (T1), 6 hours after extubation (T2), 12 hours after extubation (T3), and 24 hours after extubation (T4). Secondary outcomes included emergence time, total sevoflurane consumption, total remifentanil dose, and antiemetic use and timing. In younger children who were unable to report nausea verbally, PONV assessment was based on observable clinical signs such as retching, vomiting, increased salivation, feeding refusal, and behavioral distress, as well as nursing observations and parental reports.

### Antiemetic use and timing

Rescue antiemetic treatments administered within the first 24 postoperative hours were recorded. Rescue antiemetic administration followed predefined criteria (vomiting or a PONV score ≥ 2). Ondansetron was used as the first-line rescue antiemetic at an intravenous dose of 0.1 mg/kg (maximum dose 4 mg), in accordance with institutional clinical practice. The type of rescue antiemetic agent, timing of administration, and total number of doses were obtained from anesthesia and nursing records. Outcomes were reported as the proportion of patients requiring rescue antiemetic therapy.

### Sample size calculation

Sample size calculation was based on a previous randomized controlled study evaluating the effect of BIS-guided anesthesia on PONV incidence in children, which reported PONV rates of 53% in the control group and 17% in the BIS group. With a 95% confidence level (α = 0.05) and 80% power (1−β = 0.80), a minimum of 52 patients was required. Considering a potential dropout rate of 10%, the target sample size was set at 58 patients.^14^

### Statistical analysis

Statistical analyses were performed using IBM SPSS Statistics for Windows, version 27.0 (IBM Corp., Armonk, New York). Descriptive statistics were presented as mean ± standard deviation, median (minimum–maximum), and frequencies and percentages (n, %). Data distribution was assessed using the Kolmogorov–Smirnov test.

For normally distributed independent continuous variables, Student's t-test was used, and results are expressed as mean ± standard deviation. For non-normally distributed independent continuous variables, the Mann–Whitney U test was applied, and results are reported as median (minimum–maximum). Independent categorical variables were analyzed using Pearson's chi-square test with Yates’ continuity correction, and categorical data are presented as frequencies and percentages. A p value < 0.05 was considered statistically significant.

To further evaluate the primary outcome of 24-hour PONV incidence, binary logistic regression analysis was performed to identify independent predictors of PONV. Initially, univariable logistic regression analyses were conducted for potential risk factors including age, height, weight, sex, patient group, total doses of propofol, rocuronium, fentanyl, and remifentanil, total sevoflurane consumption, mean BIS values, ASA scores, and duration of surgery. Variables with a p value < 0.20 in univariable analysis were entered into the multiple model. The final model was determined using the backward likelihood ratio method, in which only patient group remained significant; results are presented in Table 5. A p value < 0.05 was considered statistically significant.^15^

To reduce the risk of type I error due to multiple comparisons for secondary outcomes, Bonferroni correction was applied, and statistical significance was set at p < 0.00625 (0.05/8). All randomized patients were analyzed in the groups to which they were originally assigned (intention-to-treat analysis).

## RESULTS

A total of 58 patients were included in the study, and analyses compared the BIS-guided group (n = 29) with the control group (n = 29). Baseline demographic and clinical characteristics, including age, sex, body weight, height, and ASA physical status classification, are presented in Table 1. No significant differences were observed between the groups for these variables.

**Table 1:** Baseline characteristics

		BIS-guided group	Control group
Age (years)		5 (3–8)	6 (3–8)
Weight (kg)		19.3 ± 4.3	20 ± 3.9
Height (cm)		109 (92–130)	115 (90–130)
History of PONV		0 (0)	0 (0)
Sex			
	*Male*	13 (44.8)	11 (37.9)
	*Female*	16 (55.2)	18 (62.1)
ASA physical status			
	*ASA I*	26 (89.7)	24 (82.8)
	*ASA II*	3 (10.3)	5 (17.2)

Data are presented as mean ± standard deviation or median (minimum–maximum) for continuous variables and as number (%) for categorical variables. No statistical tests were performed for baseline characteristics.

No statistically significant differences were observed between the groups in terms of duration of surgery, mean heart rate, end-tidal CO_2_, mean arterial pressure, or total doses of propofol, rocuronium, and fentanyl (p > 0.05 for all). The mean BIS value was significantly higher in the BIS-guided group than in the control group (p < 0.001) (Table 2).

**Table 2:** Periprocedural data

	BIS-guided group	Control group	p value*
Duration of surgery (min)	53.3 ± 4.7	53.6 ± 4.6	0.822^t^
SpO_2_ (%)	99 (97–100)	98 (97–100)	0.048^m^
Heart rate (bpm)	110 (85–125)	110 (88–124)	0.75^m^
ETCO_2_	36 (33–41)	38 (33–41)	0.106^m^
Mean arterial pressure (mmHg)	81 (72–92)	84 (73–92)	0.87^m^
Propofol dose (mg)	37.8 ± 8.8	40.5 ± 7.9	0.228^t^
Rocuronium dose (mg)	10 (5–15)	10 (9–16)	0.636^m^
Fentanyl dose (mcg)	20 (10–25)	20 (14–27)	0.132^m^
Mean BIS value	57 (48–60)	50 (40–54)	< **0.001** m *

Data are presented as number (%), mean ± standard deviation or median (minimum–maximum).

t Student's t-test.

m Mann–Whitney U test.

* p value < 0.05 was considered statistically significant.

PONV within the first 24 posmiddleerative hours was observed in 13 patients (44.8%) in the BIS-guided group and 23 patients (79.3%) in the control group. The difference between the groups was statistically significant (Relative risk [RR] = 0.565; 95% CI: 0.362–0.881; p = 0.015) (Table 3).

**Table 3:** Incidence of Posmiddleerative Nausea and Vomiting (PONV) according to time intervals

	BIS-guided group	Control group	Relative Risk (95% CI)	p value*
0–1 h PONV	5 (17.2)	17 (58.6)	0.294 (0.125–0.691)	**0.003** y ☨
1–6 h PONV	5 (17.2)	17 (58.6)	0.294 (0.125–0.691)	**0.003** y ☨
6–12 h PONV	6 (20.7)	11 (37.9)	0.546 (0.233–1.278)	0.249^y^
12–24 h PONV	4 (13.8)	6 (20.7)	0.667 (0.21–2.118)	0.728^y^
24 h PONV	13 (44.8)	23 (79.3)	0.565 (0.362–0.881)	**0.015** y *

Data are presented as number (%). Relative risks (RR) with 95% confidence intervals (CI) are reported.

y Pearson's chi-squared test with Yates's continuity correction.

* A p value < 0.05 was considered statistically significant for the primary outcome (24-hour PONV).

☨ For secondary outcomes, Bonferroni correction was applied, and a p value < 0.00625 was considered statistically significant.

Time-interval analyses showed that, during the early posmiddleerative period (0–1 hour and 1–6 hours), PONV incidence was significantly lower in the BIS-guided group than in the control group (for both intervals: RR = 0.294; 95% CI: 0.125–0.691; p = 0.003). No significant differences were observed between the groups during the later posmiddleerative periods (6–12 hours and 12–24 hours) (p > 0.00625, Bonferroni-adjusted) (Table 3).

Emergence time was significantly shorter in the BIS-guided group than in the control group (p < 0.001). Total sevoflurane consumption was also significantly lower in the BIS-guided group (p < 0.001). Remifentanil consumption showed no significant difference between the groups (p = 0.726). Although the requirement for antiemetic therapy was lower in the BIS-guided group, the difference did not reach statistical significance after Bonferroni correction (p = 0.015) (Table 4).

**Table 4:** Secondary outcomes

	BIS-guided group	Control group	p value
Emergence time (min)	7 (5–9)	11 (7–14)	< **0.001** m *
Sevoflurane consumption (mL)	20 (15–25)	26 (20–30)	< **0.001** m *
Remifentanil consumption (μg/kg)	5.35 ± 0.6	5.30 ± 0.5	0.726^t^
Antiemetic use	4 (13.8)	22 (75.9)	0.015^y^

Data are presented as number (%) or median (minimum–maximum).

y Pearson's chi-squared test with Yates's continuity correction.

m Mann–Whitney U test.

t Student's t-test.

* For secondary outcomes, Bonferroni correction was applied, and a p value < 0.00625 (0.05/8) was considered statistically significant.

In the multivariable logistic regression analysis, BIS-guided anesthesia was identified as an independent protective factor against the development of PONV (OR = 0.212; 95% CI: 0.067–0.675; p = 0.009) (Table 5).

**Table 5:** Logistic regression analysis for Posmiddleerative Nausea and Vomiting (PONV)

		PONV		Total	Univariable		Multiple	
		No	Yes		OR (95% CI)	p	OR (95% CI)	p value
Groups								
	*BIS-guided group*	16 (72.7)	13 (36.1)	29 (50)	0.212 (0.067 – 0.675)	0.009*	0.212 (0.067 – 0.675)	**0.009** *
	*Control group*	6 (27.3)	23 (63.9)	29 (50)	Reference			
	*Age (years)*	5.1 ± 1.7	5.8 ± 1.6	5.5 ± 1.6	1.317 (0.939 – 1.849)	0.111	–	–
	*Height (cm)*	110.4 ± 11.7	113.7 ± 10.5	112.4 ± 11	1.028 (0.978 – 1.08)	0.274	–	–
	*Weight (kg)*	19.3 ± 4.2	19.9 ± 4.1	19.6 ± 4.1	1.036 (0.909 – 1.181)	0.595	–	–
Sex								
	*Male*	7 (31.8)	17 (47.2)	24 (41.4)	1.917 (0.632 – 5.82)	0.251	–	–
	*Female*	15 (68.2)	19 (52.8)	34 (58.6)	Reference			
ASA physical status								
	*ASA I*	18 (81.8)	32 (88.9)	50 (86.2)	0.996 (0.887 – 1.119)	0.946	–	–
	*ASA II*	4 (18.2)	4 (11.1)	8 (13.8)	Reference			
	*Propofol dose (mg)*	38.3 ± 8.7	39.7 ± 8.4	39.2 ± 8.4	1.021 (0.958 – 1.088)	0.523	–	–
	*Rocuronium dose (mg)*	10.9 ± 2.6	11.7 ± 2.4	11.4 ± 2.5	1.14 (0.915 – 1.42)	0.242	–	–
	*Fentanyl dose (*μ*g*)	18.1 ± 4.2	19.2 ± 4	18.8 ± 4.1	1.065 (0.933 – 1.217)	0.352	–	–
	*Sevoflurane consumption (mL)*	21.7 ± 3.3	23.7 ± 3.5	22.9 ± 3.6	1.185 (1.005 – 1.397)	0.044*	–	–
	*Remifentanil dose (*μ*g/kg)*	5.36 ± 0.6	5.31 ± 0.6	5.33 ± 0.6	0.886 (0.366 – 2.231)	0.766	–	–
	*Mean BIS value*	54.4 ± 4.8	52.4 ± 4	53.1 ± 4.4	0.893 (0.783 – 1.019)	0.094	–	–
	*Duration of surgery (min)*	53.5 ± 5.4	53.4 ± 4.1	53.4 ± 4.6	0.996 (0.887 – 1.119)	0.946	–	–
	*Constant*	–	–	–	–	–	1.765 (0–0)	**0.055** *

Cox and Snell R Square = 0.122; Nagelkerke R Square = 0.166; Accuracy = 0.672. Data are presented as number (%) and mean ± standard deviation.

* p < 0.05 was considered statistically significant.

### Harms

No serious adverse events were observed during the study period.

## DISCUSSION

This randomized controlled trial demonstrated that, compared with a non–BIS-guided approach, BIS-guided anesthesia management was associated with a reduced incidence of PONV within the first 24 posmiddleerative hours. This effect was particularly pronounced during the early posmiddleerative period (0–1 hour and 0–6 hours). In the multivariable analysis, BIS-guided anesthesia was identified as an independent protective factor against the development of PONV. This finding corresponds to an absolute risk reduction of approximately 34.5%, suggesting a potentially clinically meaningful reduction in PONV.

PONV remains one of the most common posmiddleerative adverse events in pediatric patients and is strongly associated with the use of inhalational anesthesia.^14^ Apfel et al.^13^ demonstrated that the association between inhalational anesthesia and PONV is especially prominent during the early posmiddleerative period (0–2 hours). Previous studies on BIS-guided anesthesia in adult populations have reported a reduction in PONV incidence with BIS monitoring, likely due to more precise and controlled titration of inhalational anesthetics.^16,17^ In contrast, a recent pediatric study reported that BIS-guided inhalational anesthesia did not significantly reduce PONV during the very early posmiddleerative period (0–1 hour) but resulted in a marked reduction during the 1–24 hour period.^14^

Our findings are generally consistent with the published literature and further support the beneficial effect of BIS monitoring on PONV. Notably, unlike previous pediatric studies, our results show that BIS-guided anesthesia was also associated with a significant reduction in PONV during the immediate early posmiddleerative period (0–1 hour). This difference may be attributable to variations in surgical type, anesthetic management protocols, and posmiddleerative observation periods.

Liao et al.^18^ reported no significant difference in PONV incidence between BIS-guided and non–BIS-guided sevoflurane anesthesia in patients undergoing ambulatory surgery. Although sevoflurane consumption was lower in the BIS-guided group, PONV rates were similar between groups. This discrepancy may be explained by the inclusion of ambulatory urological procedures and the limited posmiddleerative follow-up period, which was limited to the first posmiddleerative hour. In contrast, our study focused on patients undergoing adenoidectomy, a population known to be at higher risk for PONV, and included a longer posmiddleerative observation period. Therefore, differences in surgical type and follow-up duration may account for the divergent findings. Taken together, the available evidence suggests that the effect of BIS-guided anesthesia on PONV may vary according to surgical population, anesthetic protocol, posmiddleerative observation duration, and the use of prophylactic antiemetics.

The incidence of PONV is proportional to both the duration and total dose of inhalational anesthetics administered.^19^ In the present study, surgical durations were comparable between groups; however, total sevoflurane consumption was significantly lower in the BIS-guided group. Another notable finding was the significantly shorter emergence time in the BIS-guided group. These secondary outcomes are consistent with findings from previous meta-analyses and comparable studies, which have demonstrated that BIS-guided anesthesia can reduce anesthetic consumption and accelerate recovery.^8,9,16^

In a large cohort study, Portnoy et al.^5^ evaluated the incidence of PONV, prophylactic strategies, and adherence to clinical guidelines in pediatric patients. The authors reported lower PONV rates than those commonly cited in the literature, largely attributable to the routine use of antiemetic prophylaxis. In our study, routine antiemetic prophylaxis was intentionally omitted to specifically evaluate the isolated effect of BIS-guided anesthesia on PONV. The observed PONV incidence was 44% in the BIS-guided group and 79% in the non–BIS-guided group. The relatively higher PONV incidence in the BIS-guided group compared with that in the study by Portnoy et al.^5^ may be explained by the absence of routine pharmacological prophylaxis. Although the requirement for rescue antiemetic therapy was numerically lower in the BIS-guided group, this difference did not remain statistically significant after Bonferroni correction.^20^ Nevertheless, the observed numerical reduction may still be clinically relevant and should be interpreted cautiously given the relatively small sample size.

Taken together, our findings and the published literature suggest that BIS-guided anesthesia may represent a valuable strategy for PONV prevention. The results of this study, obtained without routine antiemetic prophylaxis, show that BIS-guided sevoflurane anesthesia significantly reduces the incidence of PONV in pediatric patients undergoing adenoidectomy. BIS-guided anesthesia may therefore be considered a useful adjunctive strategy for reducing PONV in pediatric surgeries associated with a high risk of PONV. Future multicenter studies with larger sample sizes and standardized antiemetic prophylaxis protocols are warranted to further validate these findings.

### Limitations

This study has certain limitations. First, it was conducted at a single center with a relatively small sample size, which may reduce statistical power and limit the generalizability of the findings to broader pediatric surgical populations. In particular, the relatively small sample size may have increased the risk of type II error for certain secondary outcomes, including rescue antiemetic use. Second, the study population was restricted to pediatric patients undergoing adenoidectomy; therefore, the results may not be applicable to other surgical procedures or broader pediatric age groups. Third, blinding of the attending anesthesiologists was not feasible because of the BIS-guided anesthesia protocol, which may have introduced performance bias. Fourth, the absence of routine antiemetic prophylaxis may limit the applicability of the findings to routine clinical practice, although this approach allowed clearer evaluation of the isolated effect of BIS-guided anesthesia on PONV. Finally, PONV assessment was based partly on clinical observation, nursing assessments, and parental reports, particularly in younger children unable to verbalize nausea reliably; therefore, some degree of subjective assessment bias and interobserver variability cannot be excluded.

## CONCLUSIONS

This prospective randomized controlled trial demonstrated that BIS-guided sevoflurane anesthesia was associated with a reduced incidence of PONV within the first 24 posmiddleerative hours in pediatric patients undergoing adenoidectomy. BIS monitoring was also associated with lower sevoflurane consumption and shorter emergence time. These findings suggest that BIS-guided anesthesia may help optimize anesthetic titration and improve early posmiddleerative recovery in pediatric patients at high risk for PONV. However, larger multicenter studies are needed to confirm these findings and evaluate their applicability in routine clinical practice.

## Data Availability

The datasets generated and analyzed during the current study are available from the corresponding author upon reasonable request. The data are not publicly available because of privacy and ethical restrictions concerning pediatric patient information.

## References

[1] Jin Z, Gan TJ, Bergese SD (2020). Prevention and Treatment of Postoperative Nausea and Vomiting (PONV): A Review of Current Recommendations and Emerging Therapies. TCRM.

[2] Frelich M, Burša F, Jor O (2025). Effect of second-hand smoking on the incidence of postoperative nausea and vomiting in children after adenoidectomy: a single-centre retrospective study. J Anesth Analg Crit Care.

[3] Liang D, Shan Y, Wang L (2020). The effect of prophylactic rewarming on postoperative nausea and vomiting among patients undergoing laparoscopic hysterectomy: a prospective randomized clinical study. Sao Paulo Med J.

[4] Kovac AL (2021). Postoperative Nausea and Vomiting in Pediatric Patients. Pediatr Drugs.

[5] Portnoy Y, Glebov M, Orkin D, Katsin M, Berkenstadt H (2025). Postoperative Nausea and Vomiting in Pediatrics: Incidence and Guideline Adherence—a Retrospective Cohort Study. Anesthesia & Analgesia.

[6] Gan TJ, Belani KG, Bergese S (2020). Fourth Consensus Guidelines for the Management of Postoperative Nausea and Vomiting. Anesthesia & Analgesia.

[7] Talih G, Yüksek A, Şahin E (2020). Evaluation of emergence agitation after general anaesthesia in rhinoplasty patients: Inhalation anaesthesia versus total intravenous anaesthesia. American Journal of Otolaryngology.

[8] Oliveira DAC, Santos Monteiro J, Macêdo NR, Tavares Costa Santos AH, Rosa Neves SM (2025). Bispectral index-guided anesthesia in children: A systematic review and meta-analysis. Anaesthesia Critical Care & Pain Medicine.

[9] Templeton TW, Alex G, Eloy JD, Stollings L (2025). BIS Guided Titration of Sevoflurane in Pediatric Patients Undergoing Elective Surgery: A Randomized Controlled Trial. Pediatric Anesthesia.

[10] Gu Y, Hao J, Wang J, Monaco F (2024). Anesthesiology Research and Practice.

[11] Sargin M, Uluer MS, Şimşek B (2019). The effect of bispectral index monitoring on cognitive performance following sedation for outpatient colonoscopy: a randomized controlled trial. Sao Paulo Med J.

[12] Hopewell S, Chan AW, Collins GS (2025). CONSORT 2025 statement: updated guideline for reporting randomised trials. BMJ.

[13] Apfel CC, Stoecklein K, Lipfert P (2005). PONV: A problem of inhalational anaesthesia?. Best Practice & Research Clinical Anaesthesiology.

[14] Frelich M, Sklienka P, Romanová T (2024). The effect of BIS-guided anaesthesia on the incidence of postoperative nausea and vomiting in children: a prospective randomized double-blind study. BMC Anesthesiol.

[15] Gunel B, Sencan A, Cevik T, Islamoglu Z, Yilmaz M (2026). Comparison of high flow nasal cannula versus procedural oxygen mask for hypoxemia prevention during endoscopic retrograde cholangiopancreatography: a randomized parallel-group trial. Minerva Anestesiol.

[16] Oliveira CRD, Bernardo WM, Nunes VM (2017). Benefit of general anesthesia monitored by bispectral index compared with monitoring guided only by clinical parameters. Systematic review and meta-analysis. Brazilian Journal of Anesthesiology (English Edition).

[17] Nelskylä KA, Yli-Hankala AM, Puro PH, Korttila KT (2001). Sevoflurane Titration Using Bispectral Index Decreases Postoperative Vomiting in Phase II Recovery After Ambulatory Surgery. Anesthesia & Analgesia.

[18] Liao WW, Wang JJ, Wu GJ, Kuo CD (2011). The effect of cerebral monitoring on recovery after sevoflurane anesthesia in ambulatory setting in children: A comparison among bispectral index, A-line autoregressive index, and standard practice. Journal of the Chinese Medical Association.

[19] Bannister CF, Brosius KK, Sigl JC, Meyer BJ, Sebel PS (2001). The Effect of Bispectral Index Monitoring on Anesthetic Use and Recovery in Children Anesthetized with Sevoflurane in Nitrous Oxide. Anesthesia & Analgesia.

[20] Talih G, Yüksek A (2021). Septorinoplasti Hastalarında Postoperatif Analjezik Ajan Tercihleri: Retrospektif Analiz. Ankara Eğitim ve Araştırma Hastanesi Tıp Dergisi.

